# Role of Palladin Phosphorylation by Extracellular Signal-Regulated Kinase in Cell Migration

**DOI:** 10.1371/journal.pone.0029338

**Published:** 2011-12-28

**Authors:** Eri Asano, Masao Maeda, Hitoki Hasegawa, Satoko Ito, Toshinori Hyodo, Hong Yuan, Masahide Takahashi, Michinari Hamaguchi, Takeshi Senga

**Affiliations:** 1 Division of Cancer Biology, Nagoya University Graduate School of Medicine, Nagoya, Aichi, Japan; 2 Department of Respiratory Medicine, Nagoya University Graduate School of Medicine, Nagoya, Aichi, Japan; 3 Department of Obstetrics and Gynecology, Nagoya University Graduate School of Medicine, Nagoya, Aichi, Japan; 4 Department of Pathology, Nagoya University Graduate School of Medicine, Nagoya, Aichi, Japan; University of Louisville, United States of America

## Abstract

Phosphorylation of actin-binding proteins plays a pivotal role in the remodeling of the actin cytoskeleton to regulate cell migration. Palladin is an actin-binding protein that is phosphorylated by growth factor stimulation; however, the identity of the involved protein kinases remains elusive. In this study, we report that palladin is a novel substrate of extracellular signal-regulated kinase (ERK). Suppression of ERK activation by a chemical inhibitor reduced palladin phosphorylation, and expression of active MEK alone was sufficient for phosphorylation. In addition, an *in vitro* kinase assay demonstrated direct palladin phosphorylation by ERK. We found that Ser77 and Ser197 are essential residues for phosphorylation. Although the phosphorylation of these residues was not required for actin cytoskeletal organization, we found that expression of non-phosphorylated palladin enhanced cell migration. Finally, we show that phosphorylation inhibits the palladin association with Abl tyrosine kinase. Taken together, our results indicate that palladin phosphorylation by ERK has an anti-migratory function, possibly by modulating interactions with molecules that regulate cell migration.

## Introduction

The actin cytoskeleton plays pivotal roles for many fundamental processes, such as cell migration and cell division. These processes are accompanied with dynamic remodeling of the actin cytoskeleton, which is regulated by various actin-binding proteins. Extracellular stimuli such as growth factors and integrin engagement activate protein kinases, including MAPK, Src and AKT [1]. These kinases phosphorylate actin-binding proteins to control rearrangement of the actin cytoskeleton [2]. Identification of the actin-binding proteins that are phosphorylated by these kinases is essential to elucidate the molecular mechanisms by which extracellular stimuli regulate cell migration and shape changes.

Palladin, myotilin and myopalladin are a family of closely related actin-binding proteins that are expressed in a variety of muscle and non-muscle cells [2]. Among these proteins, palladin is the most abundantly expressed molecule in diverse tissues and cell lines. There are three major isoforms of palladin with apparent molecular masses of 90, 140 and 200 kDa that have proline-rich sequences and multiple IgC2 (immunoglobulin C2- type) domains [3]. Palladin is localized on actin-based subcellular structures, e.g., stress fibers, focal adhesions and podosomes [4]–[7]. Palladin has a number of associating proteins, including alpha-actinin [8], CLP36 [9] and other molecules that might affect actin organization. This implies palladin may function as a scaffolding molecule to recruit proteins to the actin cytoskeleton [6], [10]–[14]. In addition, palladin directly associates with F-actin to induce the bundling of actin filaments [15].

Accumulating evidence has shown that palladin is essential for remodeling of the actin cytoskeleton to control cell migration and invasion. Suppression of palladin expression in fibroblasts by antisense transfections results in a disruption of actin cytoskeletal organization [5]. In addition, fibroblasts derived from palladin-deficient mice show disruptions in cell motility, adhesion, and actin organization [16], [17]. Conversely, palladin overexpression in Cos7 cells and astrocytes increases the number and size of actin bundles [11], [18]. Palladin is also required for the invasion of breast cancer cells. Palladin is highly expressed in invasive breast cancer cells, and suppression of palladin expression reduces cell invasion [7]. Recent studies have shown that AKT1, which is a protein kinase essential for cell survival and cancer progression, phosphorylates palladin to regulate actin bundling and cell migration [19]. Although these studies indicate an essential role for palladin in cell migration and invasion, the precise mechanisms still remain unclear.

Extracellular signal-regulated kinase (ERK) is one of the essential molecules for the regulation of diverse cellular events including proliferation, migration, differentiation and survival [20], [21]. ERK is activated in response to various extracellular stimuli through the Ras-Raf-MEK pathway and then translocates into the nucleus to phosphorylate transcription factors [22]. Activated ERK also translocates to focal adhesions to regulate the formation of actin filaments and focal adhesions required for cell morphogenesis and migration [23]. Previous studies have demonstrated that ERK phosphorylates proteins, e.g., myosin light chain kinase [24], vinexin [25], paxillin [26], focal adhesion kinase [27], Eplin [28] and actopaxin [29], to regulate cell migration.

Palladin is a known phosphoprotein, but the identities of the protein kinases that are responsible for its phosphorylation remain uncertain. In this study, we show evidence that palladin is a novel ERK substrate. In addition, we show that palladin phosphorylation by ERK is involved in cell migration and an association with Abl tyrosine kinase.

## Materials and Methods

### Ethics statement

Use of a rabbit to produce anti-palladin antibody was approved by Committee of Animal Experiment in Nagoya University Graduate School of Medicine (Approved ID: 23130).

### Cell culture, antibodies and chemicals

Cells except MCF10A cells [30] were cultured in DMEM (Sigma, St. Louis, MO) supplemented with 10% FBS and antibiotics. MCF10A cells were maintained in DMEM-F12 (Invitrogen, Carlsbad, CA) supplemented with 0.1 µg/ml cholera toxin (Sigma), 0.02 µg/ml epidermal growth factor (PeproTech, Rocky Hill, NJ), 10 µg/ml insulin (Sigma), 0.5 µg/ml hydrocortisone (Sigma), and antibiotics. To produce an anti-palladin antibody, amino acids 705–772 of palladin were fused with GST for bacterial production, and recombinant protein was purified using glutathione agarose (Sigma). The protein was mixed with Freund's adjuvant (Sigma) and injected into a rabbit four times. The serum was then obtained. To purify the anti-palladin antibody, we used a HiTrap NHS-activated HP column (GE Healthcare BioSciences, Uppsala, Sweden) coupled with recombinant GST-palladin (705–772). Anti-HA antibody was obtained from Roche (Basel, Switzerland), anti-ERK, anti-phospho-tyrosine (PY20) and anti-Myc (9E10) antibodies were from Santa Cruz Biotechnology (Santa Cruz, CA), anti-GFP antibody was from Nacalai Tesque (Tokyo, Japan), anti-MPM2 antibody was from Millipore (Billerica, MA), anti-Rac, anti-Cdc42 and anti-Abl antibodies were from BD Biosciences (San Jose, CA), and anti-phospho-ERK antibody was from Cell Signaling (Tokyo, Japan). Epidermal Growth Factor (EGF) and Platelet Derived Growth Factor (PDGF) were purchased from PeproTech (Rocky Hill, NJ). U0126 and LY294002 were obtained from Wako (Osaka, Japan), and STI571 was from Cayman chemical (Ann Arbor, MI).

### Generation of palladin and mutant constructs

Palladin was amplified by PCR using the cDNA clone KIAA0992, which was kindly provided by Kazusa DNA Research Institute. The palladin constructs were numbered by amino acids based on the previously published sequence [4]. To produce palladin deletion constructs, DNA fragments were amplified by PCR and cloned into the pcDNA3.1 vector (Invitrogen). Amino acid substitutions were performed by PCR as previously described [31] and each mutation was confirmed by sequencing.

### Alkaline phosphatase treatment

Cells were lysed in TNE buffer (Tris-HCl 25 mM (pH7.4), NaCl 150 mM, 1% NP40) containing a cocktail of phosphatase inhibitors (Nacalai Tesque, Tokyo, Japan). Endogenous and HA-tagged palladin were immunoprecipitated with anti-palladin and anti-HA antibody, respectively. Immunoprecipitates were either not treated or treated with 10 units of alkaline phosphatase (New England Biolabs, Ispwich, MA) at room temperature for 30 min and subjected to western blot analysis.

### Generation of cell lines

To establish MDA-MB-231 [32] and MCF10A cell lines that constitutively expressed GFP, GFP-wt (GFP-tagged wild-type palladin) and GFP-S77.197G (GFP-tagged mutant palladin), each cDNA was cloned into a pQCXIN retrovirus vector (Clontech, Mountain View, CA). Silent mutations were introduced in GFP-wt and GFP-S77.197G palladin to be resistant to shRNA targeting endogenous palladin. Each plasmid was transfected into HEK 293T cells [30] together with pVPack-GP and pVPack-Ampho vectors (Stratagene, Tokyo, Japan) using Lipofectamine 2000 (Invitrogen) according to the manufacturer's protocol. Culture supernatants were collected 48 h after transfection and applied to MDA-MB-231 and MCF10A cells in combination with 2 µg/ml polybrene (Sigma). Cells were cultured for 24 h, and infected cells were selected with 400 µg/ml of G418 (Nacalai Tesque, Tokyo, Japan). To establish shLuc/GFP, shPal/GFP, shPal/GFP-wt and shPal/GFP-S77.197G cells, oligonucleotides encoding shRNAs specific for human palladin (5′-GCACAAAGGATGCTGTTAT-3′) and luciferase (5′-CTTACGCTGAGTACTTCGA-3′) were cloned into the pSIREN-RetroQ retroviral vector (Clontech). Cells were infected with recombinant retroviruses that encoded each shRNA and were selected with 1 µg/ml puromycin. To produce 293T/HA-wt cells, we cloned full-length palladin (amino acids 1–772) cDNA into a pQCXIP retrovirus vector with an N-terminal 4× HA-tag (Clontech). HEK 293T cells were infected with the recombinant virus and selected with 1 µg/ml puromycin.

### Western blot

Cell lysates were loaded on SDS-polyacrylamide gels for electrophoresis and transferred to polyvinylidene difluoride membranes (Millipore). Membranes were blocked with 1% skim milk for 1 h, incubated with primary antibodies for 1 h, washed with TBS-T for 15 min and incubated with HRP-labeled secondary antibodies. Signals were detected with ECL system (GE Healthcare BioSciences). Signal intensities were measured using Light Capture II equipped with CS analyzer (ATTO Corp., Tokyo, Japan). To detect palladin mobility shifts, serum-starved cells were stimulated with EGF (20 ng/ml) or PDGF (10 ng/ml) for 5 min and lysed with SDS sample buffer. To detect mobility shifts of exogenously expressed palladin, cells were transfected with mutant palladin. Twenty-four hours later, cells were serum-starved and stimulated with EGF (20 ng/ml) for 5 min.

### In vitro kinase assay

A GST-fused palladin fragment (amino acids 45–249) was purified from bacteria under native conditions. Purified active ERK was purchased from Millipore (Billerica, MA). One nanogram of active ERK was incubated with 1 µg of wild-type or mutant GST-palladin (45–249) bound to glutathione beads in the presence of [γ^32−^P]-ATP for 10 min. The reaction mixture was washed three times with PBS. Precipitates were separated by SDS-PAGE and analyzed by autoradiography.

### Pull-down assay

To produce GST-fused proteins, cDNAs encoding fragments of each protein were cloned into a 5X-1 vector (GE Healthcare BioSciences) at the *EcoR*I-*Not*I site. GST-fused proteins were produced in bacteria and purified using glutathione agarose. To examine the association of palladin with GST-fused proteins, serum-starved 293T/HA-wt or 293T/HA-S77.197G cells were treated or not treated with EGF (20 ng/ml) for 5 min and then lysed with TNF buffer. After centrifugation at 15,000 rpm for 10 min, supernatants were mixed with GST-fused proteins bound to glutathione agarose. Affinity precipitated proteins were separated by SDS-PAGE, transferred to PVDF membrane and probed with anti-Myc antibody.

### Migration assay

Cells were serum-starved for 2 h and suspended in serum-free media after trypsin inactivation. Cells (5×10^4^) in 200 µl were loaded onto the upper surface of a Boyden chamber with EGF (20 ng/ml) in the lower chamber. Cells were allowed to migrate into the lower surface of the filter for 2 h (MDA-MB-231 cells) or 4 h (MCF10A cells) and fixed with 70% ethanol. Cells on the upper surface of the filter were removed with a cotton swab and stained with crystal violet. Cells that migrated to the lower surface of the chamber were counted in five randomly selected fields and average numbers of migrated cells were calculated for each cell line. To determine the ratio of migrated cells relative to shLuc/GFP cells, average numbers of migrated cells from five independent fields were divided by the average number of migrated shLuc/GFP cells. Three independent experiments were performed. Results were compared using Student's *t* test and considered significant when *p* was <0.05. To examine cell migration in the presence of STI571, serum-starved cells were treated with the different concentrations of STI571 for 2 h and then subjected to the migration assay with EGF in the lower chamber. STI571 was added to the upper and lower chamber during cell migration. To determine cell migration in the absence of Abl expression, cells were transfected with either luciferase or Abl siRNA and 48 h later, cells were serum-starved and migration was assessed.

### siRNA transfection

siRNA against Abl and luciferase was designed and synthesized by Sigma. The sequence of the Abl siRNA is 5′-GGGUGUACCAUUACAGGAUTT-3′ and the luciferase siRNA is 5′-CUUACGCUGAGUACUUCGATT-3′. Cells were transfected with 20 nM siRNA using Lipofectamine RNAiMAX (Invitrogen) according to the manufacturer's protocol.

### Rac and Cdc42 activity assay

Cells were lysed with pull-down lysis buffer [50 mM Tris-HCl(pH7.4), 200 mM NaCl, 1% Nonidet P-40, 10% Glycerol, 10 mM MgCl_2_, and 1 mM EDTA] and incubated with GST-PAK-PBD (residues 67–150) fusion protein bound to glutathione agarose beads for 1 h. Beads were washed with pull-down buffer for four times and then subjected to western blot with anti-Rac and anti-Cdc42 antibodies to detect active proteins bound to the beads. Total proteins were detected by immunoblotting cell lysates.

## Results

### Palladin is phosphorylated by growth factor stimulation

**Figure 1:**
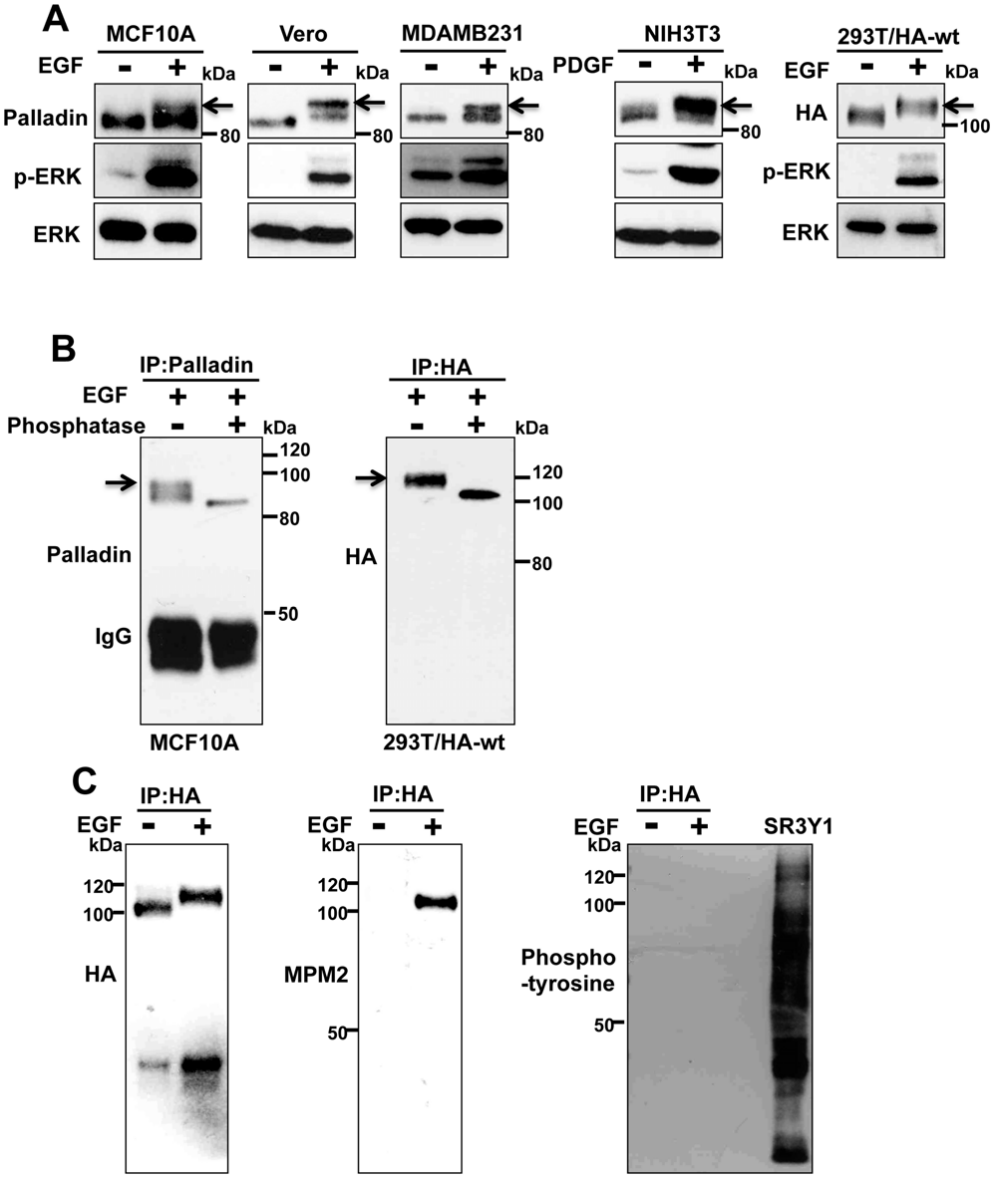
Palladin is phosphorylated upon growth factor stimulation. (A) Indicated cells were serum-starved and stimulated with EGF (20 ng/ml) or PDGF (10 ng/ml). Five min after the stimulation, cells were lysed and probed with anti-palladin or anti-HA antibody. Arrows indicate mobility shifted palladin. (B) Serum-starved MCF10A and 293T/HA-wt cells were treated with EGF and 5 min later cells were lysed with TNE buffer. The lysates were immunoprecipitated with either anti-palladin or anti-HA antibody and incubated with or without alkaline phosphatase for 30 min. Immunoprecipitates were subjected to immunoblotting with the indicated antibody. Arrows indicate mobility shifted palladin. (C) 293T/HA-wt cells were serum-starved and treated or non-treated with EGF for 5 min and then immmunoprecipitated with anti-HA antibody. The immunoprecipitates were probed with the indicated antibodies. Whole cell extract of v-Src transformed cells (SR3Y1) was used as a control for anti-phospho-tyrosine antibody (PY20).

Western blot analysis with a polyclonal palladin antibody revealed a mobility shift of palladin in cells stimulated with EGF and PDGF (Fig. 1A), which is consistent with a previous report by Goicoechea *et al*. [6]. We also examined the mobility shift of exogenously expressed palladin. Full-length palladin was amplified by PCR using cDNA clone KIAA0992 and cloned into an N-terminally 4× HA-tagged pQCXIP retrovirus vector. We established 293T cells that constitutively expressed HA-tagged palladin (293T/HA-wt) by retrovirus infection and observed the clear appearance of a slower migrating form of HA-palladin by EGF stimulation (Fig. 1A). To determine if the mobility shift was due to phosphorylation, we used alkaline phosphatase. Palladin was immunoprecipitated from the lysates of EGF-treated MCF10A or 293T/HA-wt cells and then treated with alkaline phosphatase. As shown in Figure 1B, alkaline phosphatase treatment eliminated the mobility shift of both endogenous and exogenous palladin. This mobility shift is often observed after the phosphorylation of serine or threonine residues followed by proline. To confirm the phosphorylation of serine or threonine residues of palladin, we used an anti-MPM2 antibody that recognizes a phosphorylated epitope (Ser/Thr)-Pro. As shown in Figure 1C, only a mobility-shifted form of palladin was detected by the anti-MPM2 antibody. Anti-phospho-tyrosine antibody did not react with palladin in EGF-treated cells (Fig. 1C). These results indicate that palladin is phosphorylated at serine or threonine in response to growth factor stimulation.

### MEK/ERK is responsible for palladin phosphorylation

**Figure 2:**
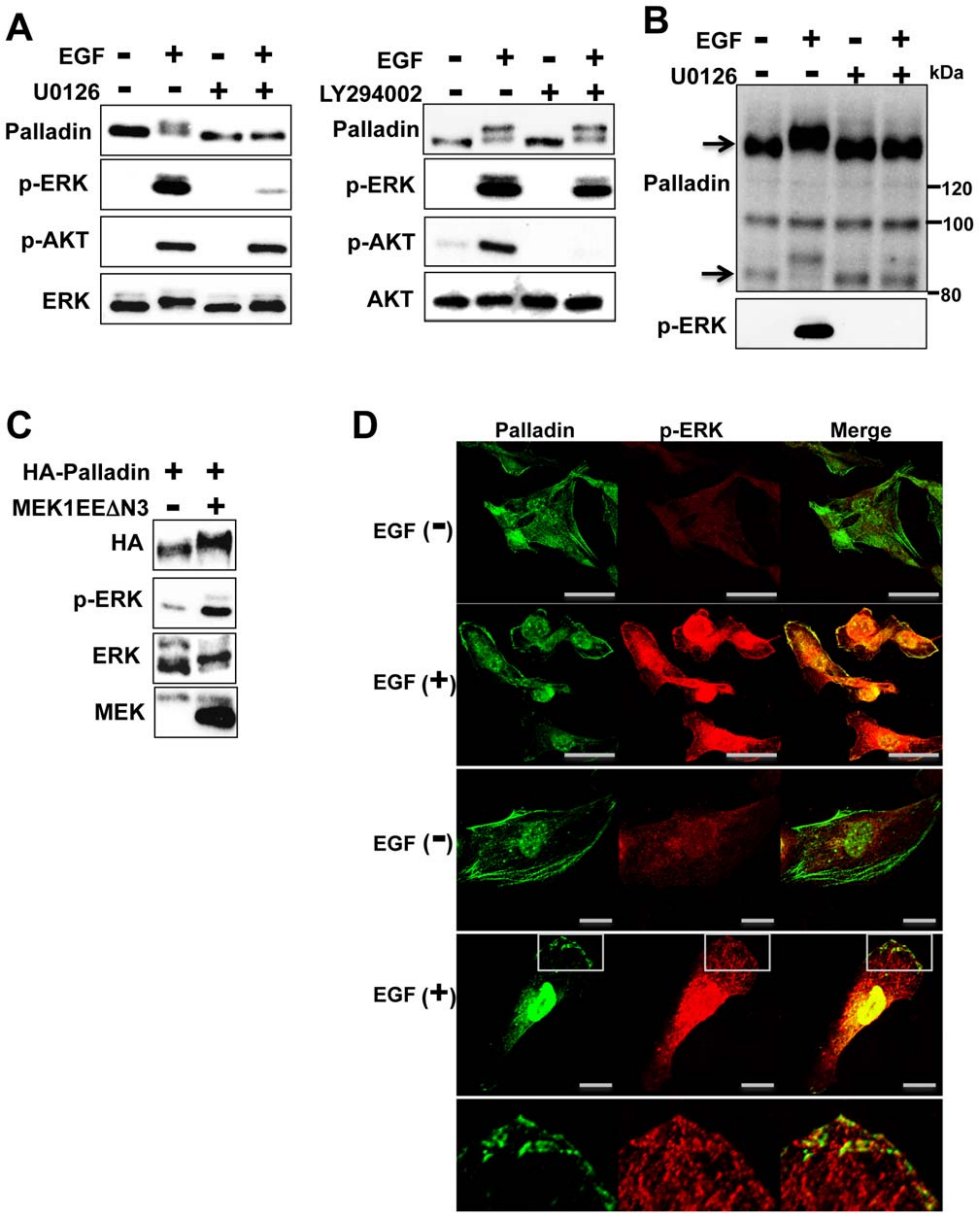
Palladin phosphorylation is dependent on the MEK/ERK pathway. (A) Serum-starved Vero cells were treated with EGF in the presence or absence of the indicated inhibitors. Five minutes after stimulation, cells were lysed and immunoblotted with anti-palladin antibody. (B) HeLa cells were treated as in (A) and immunblotted with anti-palladin antibody. Arrows indicate 90 kDa and 140 kDa form of palladin. (C) Cos7 cells were transfected with HA-palladin together with or without active MEK (MEK1/EEΔN3). Twenty-four hours later, cells were lysed and immunoblotted with the indicated antibodies. (D) MDA-MB-231 cells were cultured on glass coverslips and stimulated with EGF for 5 min and then fixed to immunostain for palladin (green) and phospho-ERK (red). Pictures were taken using an A1Rsi confocal microscopy (Nikon). Images in the bottom panel are partially enlarged images of the squared fields of the pictures. (Upper panels; scale bar = 50 µm, Lower panels; scale bar = 15 µm).

To determine which protein kinases are essential for phosphorylation, we used a MEK inhibitor (U0126) and a PI3K inhibitor (LY294002). Serum-starved cells were incubated with U0126 (5 µM) or LY294002 (10 µM) for 1 h and stimulated with EGF (20 ng/ml) for 5 min. Addition of U0126 or LY294002 clearly suppressed activation of ERK or AKT, respectively (Fig. 2A). Although the mobility shift was clearly visible in the presence of LY294002, the addition of U0126 significantly diminished the palladin gel shift (Fig. 2A). We also used HeLa cells that mainly expressed 140 kDa form of palladin. Mobility shift of 140 kDa form of palladin was inhibited by U0126 treatment as well (Fig. 2B). To further confirm that MEK/ERK was responsible for the phosphorylation, we expressed HA-palladin together with or without active MEK. As shown in Figure 2C, HA-palladin only showed a mobility shift in the presence of active MEK (MEK1EEΔN3) [33]. These results clearly indicate that MEK/ERK regulates palladin phosphorylation. We examined palladin localization and phosphorylated ERK after EGF stimulation. MDA-MB-231 cells cultured on glass coverslips were stimulated with EGF for 5 min, fixed by paraformaldehyde and then immunostained for palladin and phosphorylated ERK. As shown in Figure 2D, palladin and phosphorylated ERK colocalized to the membrane edge after EGF stimulation.

### N-terminal palladin serine residues are phosphorylated

**Figure 3:**
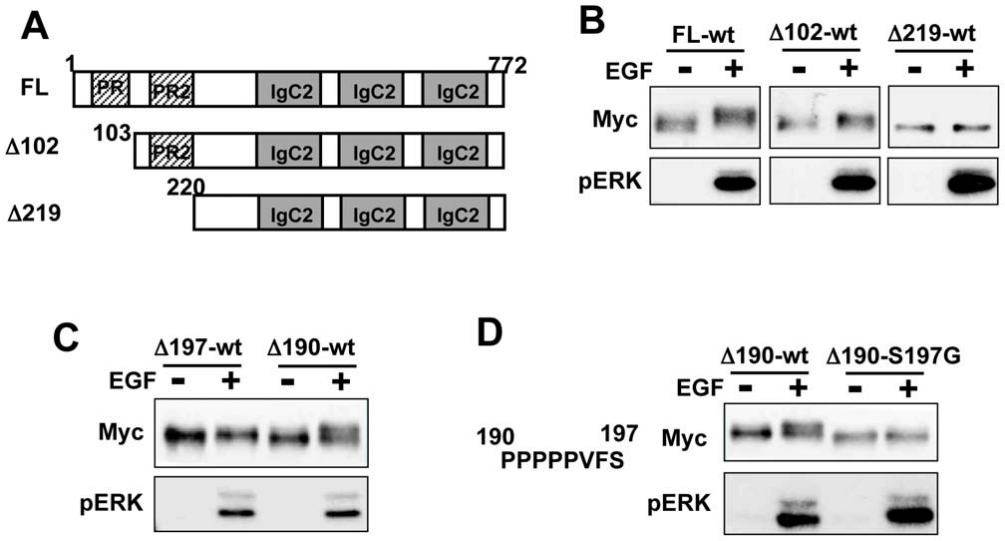
Ser197 is phosphorylated by EGF stimulation. (A) Schematic representation of palladin deletion constructs. (B) Mobility shift of mutant palladin after EGF stimulation was examined by western blot. Vero cells were transfected with Myc-tagged mutant palladin. Twenty-four hours later, serum-starved cells were stimulated with or without EGF for 5 min and lysed for western blot analysis. Lower panel shows ERK phosphorylation. (C) Mobility shift of mutant palladin after EGF stimulation was examined by western blotting. (D) Mobility shifts of Δ190-wt and Δ190-S190G after EGF stimulation were examined by western blotting. Amino acid sequence from 190–197 is indicated.

To determine which residues were phosphorylated by the EGF treatment, we deleted the N-terminus of palladin and examined its gel shift. This palladin sequence has two proline-rich regions between amino acids 65–100 and 180–220. The Δ102 and Δ219 deletions are missing the first or both proline rich regions, respectively (Fig. 3A). We transiently expressed Myc-tagged full-length, Δ102 or Δ219 palladin in Vero cells and examined mobility shifts by EGF stimulation. Although a slower migrating form of Δ102 appeared due to EGF stimulation, Δ219 did not show any mobility shift (Fig. 3B). To narrow down the phosphorylated region, we created Δ197 and Δ190, i.e., amino acids 1–197 and 1–190 were deleted, respectively. As shown in Figure 3C, a mobility shift was only observed for Δ190 and not for Δ197. There is only one serine residue (Ser197) in this region. Therefore, we mutated this residue to glycine (S197G) in Δ190 and examined the gel shift. EGF treatment did not induce a Δ190-S197G mobility shift, which suggested that Ser197 is phosphorylated in response to EGF (Fig. 3D).

**Figure 4:**
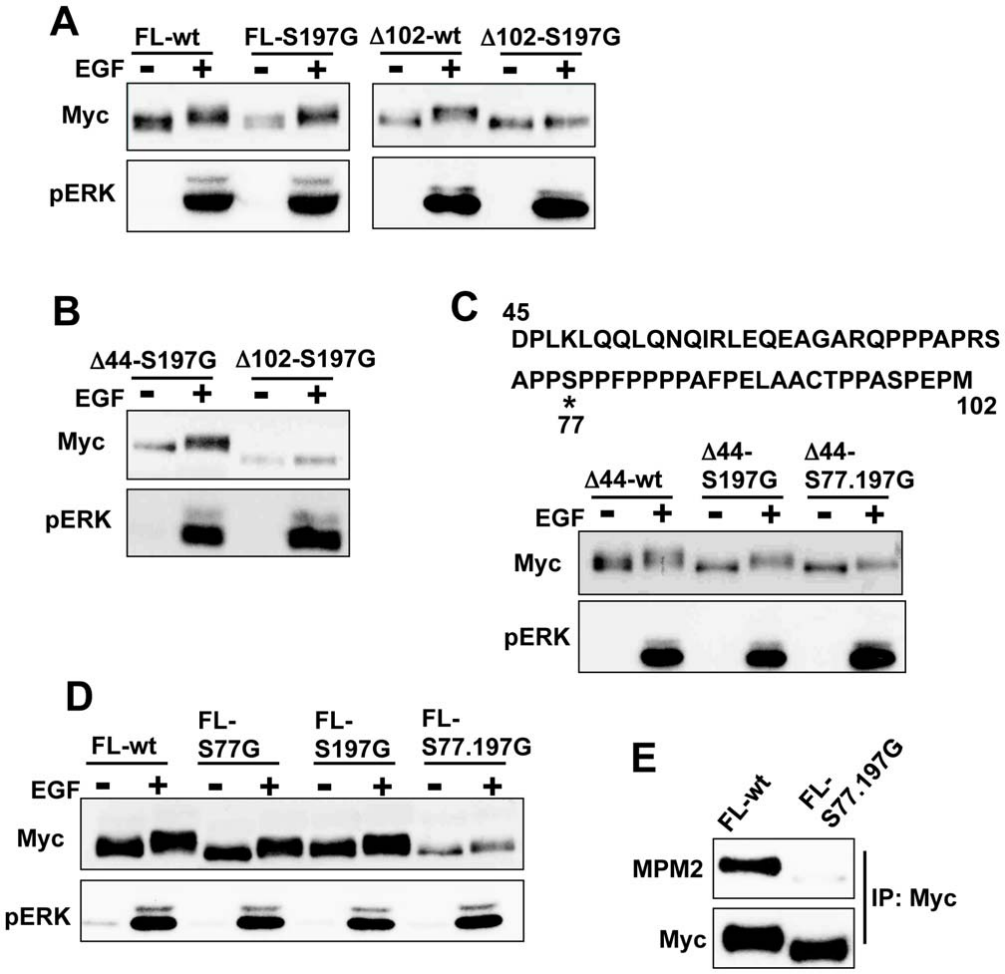
Ser77 is phosphorylated by EGF stimulation. (A) Vero cells were transfected with the indicated mutant palladin. Twenty-four hours later, serum-starved cells were stimulated with EGF for 5 min. Mobility shifts were examined by western blotting. (B) Mobility shifts of Δ44-S197G and Δ102-S197G were examined by western blotting. (C) Amino acid sequence from 45 to 102 is indicated. Mobility shift of the indicated mutant palladin was examined by western blotting. (D) Mobility shift of the indicated mutant palladin was examined by western blotting. (E) Vero cells were transfected with either FL-wt or FL-S77.197G palladin. Twenty-four hours later, cells were stimulated with EGF for 5 min. Cells were lysed and immunoprecipited with anti-Myc antibody and blotted with anti-MPM2 antibody.

To investigate whether there are other residues that are phosphorylated after EGF stimulation, we substituted Ser197 in full-length and Δ102 palladin to glycine (FL-S197G and Δ102-S197G). FL-S197G, but not Δ102-S197G, showed a mobility shift due to EGF stimulation (Fig. 4A), which suggested there are other phosphorylated residues in the N-terminal region. Deletion of 44 amino acids in the N-terminus did not eliminate the palladin gel shift in the S197G mutant (Δ44-S197G) (Fig. 4B). We used ScanSite [34] to search for candidate residues for phosphorylation between residues 45–102 and found that Ser77 was most likely to be phosphorylated by ERK. Substitution of both Ser77 and Ser197 in Δ44-palladin (Δ44-S77.197G) clearly reduced the mobility shift compared to Δ44-S197G (Fig. 4C). We also used full-length palladin with substitutions of both serine residues (FL-S77.197G). As shown in Figure 4D, the mobility shift of FL-S77.197G was reduced compared to both FL-S77G and FL-S197G. In addition, western blot analysis with an anti-MPM-2 antibody revealed a significant reduction of FL-S77.197G phosphorylation after EGF stimulation (Fig. 4E).

**Figure 5:**
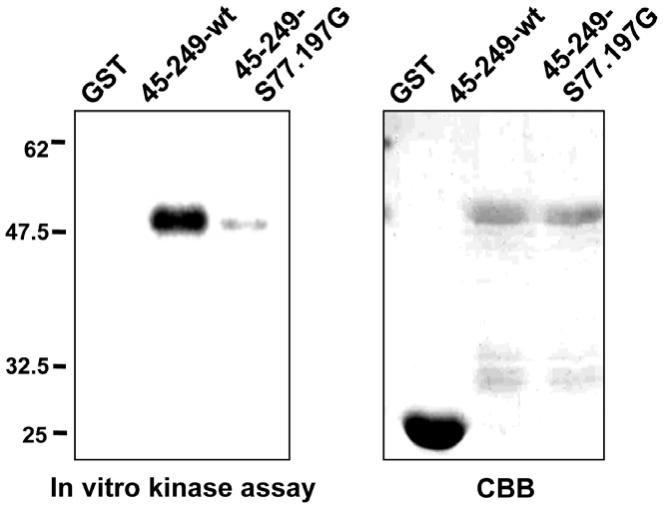
ERK phosphorylates palladin in an in vitro kinase assay. GST-fused residues 45–249 of wild-type or mutant palladin was incubated with active ERK for 10 min at 37°C in the presence of radioactive [γ^32−^P]-ATP and separated by SDS-PAGE. Left panel shows the phosphorylation of recombinant proteins, and the right panel shows Coomassie blue staining of recombinant proteins.

To confirm that ERK directly phosphorylates palladin, we performed *in vitro* kinase assays. We used GST-fused N-terminal palladin (amino acids 45–249) purified from bacteria as a substrate. As shown in Figure 5, phosphorylation of Ser77- and Ser197-substituted palladin was significantly reduced compared to wild-type palladin. Taken together, these results clearly indicate that Ser77 and Ser197 are phosphorylated by ERK.

### Palladin phosphorylation by ERK regulates cell migration

**Figure 6:**
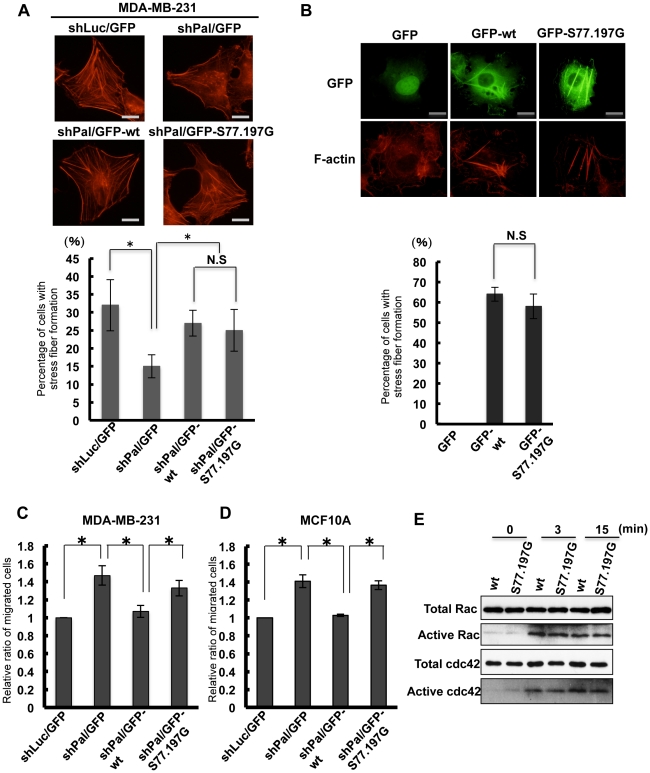
Phosphorylation of Ser77 and Ser197 controls cell migration. (A) Cells were cultured on glass coverslips for 24 h and fixed with paraformaldehyde. Cells were stained with rhodamine-conjugated phalloidin to visualize the actin cytoskeleton. (Scale bar = 15 µm) To quantitate cells with stress fiber formation, 50 cells for each cell line were evaluated and the graph shows the percentage of cells with stress fiber formation. Three independent experiments were performed. Each bar represents mean ± SD. (N.S; P>0.05, *P<0.01) (B) Cos7 cells were transfected with either GFP-wt or GFP-S77.197G palladin. Twenty-four hours later, cells were fixed and stained with rhodamine-conjugated phalloidin. (Scale bar = 15 µm) To quantitate cells with thick stress fiber formation, 30 transfected cells were evaluated and the graph shows the percentage of cells with thick stress fiber formation. Three independent experiments were performed. Each bar represents mean ± SD. (N.S; P>0.05) (C) Cell migration was examined using a modified Boyden chamber. Three independent experiments were performed, and relative ratios of migrated cells are indicated (means±SD; *P<0.01). (D) Cell migration was examined using a modified Boyden chamber. Three independent experiments were performed, and relative ratios of migrated cells are indicated (means ±SD; *P<0.01). (E) shPal/GFP-wt and shPal/GFP-S77.197G cells (MDA-MB-231) were serum-starved and stimulated with EGF for the indicated time points. Cells were lysed and incubated with GST-fused GST-PAK-PBD fusion protein bound to glutathione-agarose beads. Beads were subjected to western blot with anti-Rac or anti-Cdc42 antibody. Total Rac or cdc42 protein was detected by immunoblotting of cell lysates.

ERK activation regulates various physiological functions, including cell migration. To investigate whether palladin phosphorylation by ERK regulates cell migration, we established cell lines that expressed wild-type or mutant palladin. We first established MDA-MB-231 cells that constitutively expressed GFP, GFP-wt (GFP-tagged full-length palladin) and GFP-S77.197G (GFP-tagged full-length S77.197G palladin) using retroviruses. We then infected a retrovirus that encoded an shRNA for luciferase into GFP-expressing cells, and an shRNA for palladin into GFP, GFP-palladin and GFP-S77.197G-expressing cells. These four cell lines were designated shLuc/GFP, shPal/GFP, shPal/GFP-wt and shPal/GFP-S77.197G, respectively. Expression of endogenous palladin was significantly suppressed in shPal/GFP, shPal/GFP-wt and shPal/GFP-S77.197G cells (Fig. S1A). Both shPal/GFP-wt and shPal/GFP-S77.197G cells expressed levels of exogenous palladin comparatively higher than endogenous palladin (Fig. S1A). Consistent with previous findings, silencing of palladin expression suppressed actin stress fiber formation. Expression of either wild-type or mutant palladin recovered stress fiber formation similar to shLuc/GFP cells (Fig. 6A). Previous studies have shown that palladin overexpression induced formation of thick actin filaments [11]. To address whether ERK phosphorylation is required for this process, we transiently overexpressed wild-type or mutant palladin in Cos7 cells and observed stress fiber formation. Expression of both wild type and mutant palladin resulted in the production of thick actin stress fibers (Fig. 6B), suggesting that ERK-mediated palladin phosphorylation is not essential for the organization of stress fibers.

We tested if palladin phosphorylation was associated with dynamic actin remodeling such as production of lamellipodia. We stimulated shPal/GFP-wt and shPal/GFP-S77.197G cells with EGF and fixed 5 min later to immunostain for actin cytoskeleton. Production of lamellipodia and disruption of stress fiber upon EGF stimulation was observed in these cells. (Fig. S2). Quantification of these results suggested that palladin phosphorylation was not required for the EGF-mediated formation of lamellipodia in MDA-MB-231 cells (Fig. S2).

We next examined cell migration using a modified Boyden chamber. As shown in Figure 6C, suppression of palladin expression enhanced cell migration, but the re-expression of wild-type palladin (shPal/GFP-wt) reduced migration to levels similar to shLuc/GFP cells. Interestingly, migration of shPal/GFP-S77.197G cells was around 24% faster than shPal/GFP-wt cells. To further confirm this result, we used MCF10A cells and established the same series of cell lines (Fig. S1B). Consistent with the MDA-MB-231 cells, MCF10A cells that expressed non-phosphorylated palladin showed enhanced migration compared to wild-type palladin expressing cells (Fig. 6D). These results suggest that palladin phosphorylation by ERK has an anti-migratory function in these cell lines.

Small GTPases are central players for the regulation of cell migration. We examined whether activation of Rac and Cdc42 is related to the phosphorylation status of palladin. ShPal/GFP-wt and shPal/GFP-S77.197G cells were serum-starved and stimulated with EGF, and then active Rac and Cdc42 was affinity precipitated by GST-fused PAK-PBD. We found that both Rac and Cdc42 were similarly activated in these cells (Fig. 6E), indicating that activation of Rac and Cdc42 in response to EGF is independent of the phosphorylation of palladin by ERK. The result suggests that ERK-mediated phosphorylation of palladin utilizes different pathways other than Rac and Cdc42 to control cell migration.

### Palladin phosphorylation affects association with Abl

**Figure 7:**
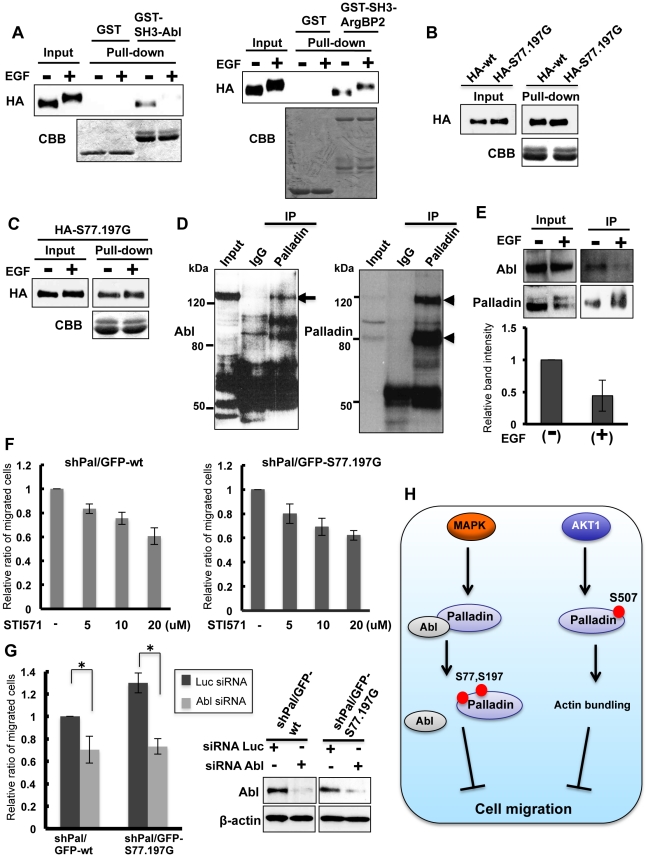
Phosphorylation of Ser77 and Ser197 regulates palladin association with Abl. (A) Serum-starved 293T/HA-wt cells were either stimulated or non-stimulated with EGF for 5 min, and cell lysates were affinity precipitated with the indicated recombinant proteins (SH3-Abl; aa66–118, SH-ArgBP2; aa432–666). Lower panels show the Coomassie blue staining of recombinant proteins. (B) HEK 293T cells were transfected with either HA-tagged palladin (HA-wt) or HA-tagged S77.197G palladin (HA-S77.197G). Twenty-four hours later, cells were lysed and affinity precipitated with GST-SH3-Abl protein bound to glutathione agarose. Immunoprecipitates were blotted with anti-HA antibody. Lower panel shows the Coomassie blue staining of the GST-SH3 Abl protein. (C) Serum-starved 293T/HA-S77.197G cells were either stimulated or non-stimulated with EGF for 5 min, and cell lysates were affinity precipitated with GST-SH3-Abl protein bound to glutathione agarose. Immunoprecipitates were blotted with anti-HA antibody. (D) MDA-MB-231 cells were lysed and immunprecipitated with either control IgG or anti-palladin antibody. The lysates were blotted with anti-Abl or anti-palladin antibody. The arrow indicates Abl and the arrowheads indicate palladin. The band around 140 kDa is an alternative form of palladin. (E) MDA-MB-231 cells were serum-starved and stimulated with or without EGF and lysed for immunopreciptation. The lyates were blotted with anti-Abl and anti-palladin antibodies. The graph shows the relative band intensity of immunoprecipitated Abl from three independent experiments. (F) Migration of shPal/GFP-wt and shPal/GFP-S77.197G cells (MDA-MB-231) was examined in the presence of indicated concentrations of STI571. Relative ratio of migrated cells was normalized by the number of migrating cells in the absence of STI571. Three independent experiments were performed and the data are shown as mean ±SD. (G) shPal/GFP-wt and shPal/GFP-S77.197G cells (MDA-MB-231) were transfected with either luciferase (Luc) or Abl siRNA and 48 h later, cell migration was examined. Relative ratio of migrated cells was normalized by the number of migrating shPal/GFP-wt cells transfected with Luc siRNA. Three independent experiments were performed and the data are shown mean ±SD (*P<0.01). Expression of Abl after siRNA transfection was examined by immunoblot. (H) Schematic presentation of regulation of cell migration by palladin phosphorylation.

To determine the molecular mechanism by which palladin phosphorylation regulates cell migration, we tested if phosphorylation affects palladin interactions with other molecules. Association of the proline rich region and the SH3 domain is essential for the signal transduction in response to extracellular stimuli. Since the phosphorylated serines are localized in the proline-rich regions, we speculated that the phosphorylation may affect association of palladin with SH3 domain. We created GST-fused SH3 for Abl and ArgBP2 and performed pull-down assays. Both proteins are known to regulate cell migration [35], [36], and a previous study has shown the association of palladin with the ArgBP2 SH3 [14]. 293T/HA-wt cells were treated with or without EGF, and cell lysates were mixed with GST-fused proteins bound to glutathione beads. Interacting proteins were affinity precipitated. As shown in Figure 7A, the palladin interaction with the Abl SH3, but not the ArgBP2 SH3, was significantly reduced by phosphorylation. We also examined the association of palladin with other known interacting proteins, e.g. profilin, alpha-actinin and CLP36, but phosphorylation did not affect these interactions (Fig. S3). If the phosphorylations of Ser77 and Ser197 were responsible for the inhibition of this interaction, the Abl SH3 would bind to S77.197G-palladin derived from EGF-treated cells. We first examined whether the substitution of Ser77 and Ser197 would affect the interaction. As shown in Figure 7B, the Abl GST-SH3 affinity precipitated HA-Ser77.197G-palladin to a level similar to wild-type palladin. We then examined the interaction of the Abl SH3 and mutant palladin in EGF-treated or non-treated cells. As shown in Figure 7C, HA-Ser77.197G-palladin in either EGF-treated or non-treated cells similarly associated with the Abl SH3.

We next tested if Abl was associated with palladin in cells. Palladin was immunopreciptiated from lysates of MDA-MB-231 cells and the immunoprecipitates were probed with anti-Abl antibody by immunoblotting. Abl was co-immunoprecipitated by anti-palladin antibody, but not by control antibody, indicating the interaction of both proteins in cells (Fig. 7D). We then examined the interaction after EGF stimulation. As shown in figure 7E, association of Abl with palladin was reduced by EGF stimulation. These results suggest that phosphorylation of Ser77 and Ser197 by ERK regulates the palladin interaction with the Abl SH3.

Finally, we examined whether Abl was essential for cell migration. Both shPal/GFP-wt and shPal/GFP-S77.197G were treated with an Abl kinase inhibitor, STI571, and the migration was examined. We found that addition of STI571 suppressed migration of both cells in a concentration dependent manner (Fig. 7F). Furthermore, silencing of Abl by siRNA transfection similarly suppressed cell migration (Fig. 7G). These results indicate a possible involvement of palladin-Abl association for EGF-mediated cell migration (Fig. 7H).

## Discussion

Since its discovery in 2000, palladin has been known as a phosphoprotein because its slower migrating form was often observed by western blotting. However, the identities of the protein kinases that phosphorylate palladin have remained elusive. A recent study has shown that AKT1 phosphorylates palladin to modulate its activity in bundling actin stress fibers and to inhibit cell migration [19]. In this report, we showed evidence that palladin is also phosphorylated by ERK in response to growth factor stimulation. Addition of MEK inhibitor clearly suppressed palladin phosphorylation upon EGF treatment. In addition, expression of active MEK alone was sufficient to phosphorylate palladin. Furthermore, *in vitro* kinase assays demonstrated direct palladin phosphorylation by active ERK. These results clearly show that palladin is a novel substrate of ERK. By making deletion constructs and point mutations, we identified Ser77 and Ser197 as residues phosphorylated by ERK. Both serine residues are conserved in the previously identified mouse palladin [5]. Mobility shifts due to EGF treatment were clearly suppressed by the substitution of both serine residues. *In vitro* kinase assays also demonstrated a significant reduction of palladin phosphorylation after the substitution of both residues. Although we cannot rule out the possibility that there are other residues that could be phosphorylated by ERK because minor phosphorylation was observed by an *in vitro* kinase assay, our results clearly show that Ser77 and Ser197 are phosphorylated by ERK.

Palladin is known as a critical factor in regulating actin cytoskeletal organization and cell migration [2]. Consistent with previous findings, palladin silencing disrupted stress fiber formation [5], which was restored by the re-expression of palladin. There have been two distinct reports regarding the role of palladin in cell migration. One report showed cell migration inhibition due to palladin knockdown [7]. The other demonstrated an anti-migratory palladin function [19]. We produced MCF10A and MDA-MB-231 cells that constitutively reduced palladin expression using shRNA and observed enhanced cell migration. Moreover, palladin re-expression reduced cell migration to levels similar to control cells, which confirmed the inhibitory palladin function in migration. Disruption of actin cytoskeleton can sometimes accelerate cell migration. For example, addition of Rho kinase inhibitor, which inhibits actin cytoskeletal organization, promotes migration of certain cell lines [37], [38]. Therefore, disruption of actin cytoskeleton may have promoted migration of palladin-knockdown cells. Although further studies are required to determine the exact function of palladin in cell migration, our results show a clear anti-migratory role for palladin in these cell lines.

A number of studies have demonstrated that ERK activation is essential for migration of numerous cell types [20]. We speculated that palladin phosphorylation is required for ERK-mediated promotion of cell migration. Contrary to our speculation, S77.197G-palladin expression in MDA-MB-231 and MCF10A cells enhanced migration compared to wild-type palladin. Suppression of ERK activation by a chemical inhibitor reduced cell migration of both the MDA-MB-231 and MCF10A cell lines (data not shown). Therefore, ERK activation is essential for the migration of these cell lines. ERK phosphorylates a variety of proteins that are involved in actin cytoskeletal remodeling to enhance migration. Most of these phosphorylations promote cell migration, but our results indicate that some ERK phosphorylations may have anti-migratory roles. ERK stimulates both activating and inhibitory signals for migration simultaneously, and ERK-mediated promotion of cell migration may be accomplished by the dominant effects of promoting signals.

To examine the molecular mechanism of palladin phosphorylation by ERK in regards to how it inhibits cell migration, we checked activation of small GTPases, Rac1 and Cdc42, which are critically involved in cell migration. We did not observe any difference in activation of both GTPases between shPal/GFP-wt and shPal/GFP-S77.197G cells. Previous studies have shown that palladin phosphorylation by AKT1 can suppress cell migration and induce bundling of actin filaments [19]. However, our results showed that palladin phosphorylation by ERK was not required for maintenance of the organized actin cytoskeleton and actin bundling. These results suggest that ERK-mediated palladin phosphorylation utilizes different molecular mechanisms to control cell migration. We speculated that phosphorylation may affect palladin associations with other molecules because phosphorylated residues are localized in proline-rich regions. We found that the association of palladin and the Abl SH3 domain was significantly reduced by phosphorylation. The association of palladin with the ArgBP2 SH3 was not affected by phosphorylation, which indicated that phosphorylation regulates interactions with specific proteins. Abl is known to promote lamellipodia formation and cell migration [35]. Therefore, Abl dissociation from palladin may inhibit Abl-mediated cell migration. However, since palladin has multiple interacting proteins, there may be other interactions regulated by phosphorylation to control cell migration.

Interactions of proline-rich regions and SH3 domains are essential for various signal transductions that regulate proliferation, differentiation, migration and transformation [39]. Although a large number of SH3-mediated interactions have been reported, little is known about the regulatory mechanisms of these interactions. SH3 and proline-rich regions form stable complexes, but our results imply that this interaction can be regulated by phosphorylation of proline-rich regions. Additional studies may reveal that SH3-mediated interactions are not stable, but reversibly regulated by phosphorylation by various protein kinases.

In summary, we have shown that palladin is a novel substrate of ERK and identified serine residues that are phosphorylated by ERK. Furthermore, we have shown that phosphorylation has anti-migratory functions and can inhibit the association of palladin with Abl. In addition to ERK and AKT1, there may be other kinases that phosphorylate palladin to regulate the actin cytoskeletal organization. Further studies are required to elucidate the molecular mechanism by which palladin regulates the integrity of the actin cytoskeleton and cell migration.

## Supporting Information

Figure S1 **Expression of endogenous and exogenous palladin in each cell line.** (A) Expression of endogenous palladin and exogenously expressed proteins in the indicated cell lines were examined by western blotting. An arrow indicates exogenously expressed palladin, and an arrowhead indicates endogenous palladin. (B) Endogenous palladin expression and exogenously expressed proteins in the indicated cell lines were examined by western blotting.
(TIF)

Figure S2 **shPal/GFP-wt and shPal/GFP-S77.197G cells were serum-starved and stimulated with EGF for 5 min.** Cells were fixed and immunostained with rhodamin-conjugated phalloidin. (Scale bar = 10 µm) The arrows indicate lamellipodia formation. The graph shows the ratio of cells with lamellipodia formation. Thirty cells were evaluated for lamellipodia formation and three independent experiments were performed. The data are shown as mean ±SD. (N.S; P>0.05).
(TIF)

Figure S3 **The phosphorylation of palladin does not affect the interaction with profilin, alpha-actinin, and CLP36.** 293T/HA-wt cells were either stimulated or non-stimulated with EGF for 5 min, and cell lysates were affinity precipitated with the indicated recombinant proteins. The precipitate were immnoblotted with anti-HA antibody. Lower panels show the Coomassie blue staining of recombinant proteins.
(TIF)

## References

[1] Porter AC, Vaillancourt RR (1998). Tyrosine kinase receptor-activated signal transduction pathways which lead to oncogenesis.. Oncogene.

[2] Otey CA, Rachlin A, Moza M, Arneman D, Carpen O (2005). The Palladin/Myotilin/Myopalladin Family of Actin-Associated Scaffolds.. Int Rev Cytol.

[3] Goicoechea SM, Arneman D, Otey CA (2008). The role of palladin in actin organization and cell motility.. Eur J Cell Biol.

[4] Mykkanen OM, Gronholm M, Ronty M, Lalowski M, Salmikangas P (2001). Characterization of Human Palladin, a Microfilament-associated Protein.. Mol Biol Cell.

[5] Parast MM, Otey CA (2000). Characterization of Palladin, a Novel Protein Localized to Stress Fibers and Cell Adhesions.. J Cell Biol.

[6] Goicoechea S, Arneman D, Disanza A, Garcia-Mata R, Scita G (2006). Palladin binds to Eps8 and enhances the formation of dorsal ruffles and podosomes in vascular smooth muscle cells.. J Cell Sci.

[7] Goicoechea SM, Bednarski B, Garcia-Mata R, Prentice-Dunn H, Kim HJ (2009). Palladin contributes to invasive motility in human breast cancer cells.. Oncogene.

[8] Ronty M, Taivainen A, Moza M, Otey CA, Carpen O (2004). Molecular analysis of the interaction between palladin and alpha-actinin.. FEBS Lett.

[9] Maeda M, Asano E, Ito D, Ito S, Hasegawa Y (2009). Characterization of interaction between CLP36 and palladin.. FEBS J.

[10] Ronty M, Taivainen A, Heiska L, Otey C, Ehler E (2007). Palladin interacts with SH3 domains of SPIN90 and Src and is required for Src-induced cytoskeletal remodeling.. Exp Cell Res.

[11] Rachlin AS, Otey CA (2006). Identification of palladin isoforms and characterization of an isoform-specific interaction between Lasp-1 and palladin.. J Cell Sci.

[12] Boukhelifa M, Parast MM, Bear JE, Gertler FB, Otey CA (2004). Palladin is a novel binding for Ena/VASP family members.. Cell Motil Cytoskeleton.

[13] Boukhelifa M, Moza M, Johansson T, Rachlin A, Parast M (2006). The proline-rich protein palladin is a binding partner for profilin.. FEBS J.

[14] Ronty M, Taivainen A, Moza M, Kruh GD, Ehler E (2005). Involvement of palladin and alpha-actinin in targeting of the Abl/Arg kinase adaptor ArgBP2 to the actin cytoskeleton.. Exp Cell Res.

[15] Dixon RD, Arneman DK, Rachlin AS, Sundaresan NR, Costello MJ (2008). Palladin Is an Actin Cross-linking Protein That Uses Immunoglobulin-like Domains to Bind Filamentous Actin.. J Biol Chem.

[16] Liu XS, Luo HJ, Yang H, Wang L, Kong H (2007). Palladin regulates cell and extracellular matrix interaction through maintaining normal actin cytoskeleton architecture and stabilizing beta1-integrin.. J Cell Biochem.

[17] Luo H, Liu X, Wang F, Huang Q, Shen S (2005). Disruption of palladin results in neural tube closure defects in mice.. Mol Cell Neurosci.

[18] Boukhelifa M, Hwang SJ, Valtschanoff JG, Meeker RB, Rustioni A (2003). Mol Cell Neurosci.

[19] Chin YR, Toker A (2010). The Actin-Bundling Protein Palladin Is an Akt1-Specific Substrate that Regulates Breast Cancer Cell Migration.. Mol Cell.

[20] Huang C, Jacobson K, Schaller MD (2004). MAP kinases and cell migration.. J Cell Sci.

[21] Johnson GL, Lapadat R (2002). Mitogen-activated protein kinase pathways mediated by ERK, JNK, and p38 protein kinases.. Science.

[22] Seger R, Krebs EG (1995). The Mapk signaling cascade.. FASEB J.

[23] Fincham VJ, James M, Frame MC, Winder SJ (2000). Active ERK/MAP kinase is targeted to newly forming cell-matrix adhesions by integrin engagement and v-Src.. EMBO J.

[24] Nguyen DH, Catling AD, Webb DJ, Sankovic M, Walker LA (1999). Myosin light chain kinase functions downstream of Ras/ERK to promote migration of urokinase-type plasminogen activator-stimulated cells in an integrin-selective manner.. J Cell Biol.

[25] Mitsushima M, Suwa A, Amachi T, Ueda K, Kioka N (2004). Extracellular signal-regulated kinase activated by epidermal growth factor and cell adhesion interacts with and phosphorylates vinexin.. J Biol Chem.

[26] Liu ZX, Yu CF, Nickel C, Thomas S, Cantley LG (2002). Hepatocyte growth factor induces ERK-dependent paxillin phosphorylation and regulates paxillin-focal adhesion kinase association.. J Biol Chem.

[27] Hunger-Glaser I, Salazar EP, Sinnett-Smith J, Rozengurt E (2003). Bombesin, lysophosphatide acid, and epidermal growth factor rapidly stimulate focal adhesion kinase phosphorylation atSer-910: requirement for ERK activation.. J Biol Chem.

[28] Han MY, Kosako H, Watanabe T, Hattori S (2007). Extracellular Signal-Regulated Kinase/Mitogen-Activated Protein Kinase Regulates Actin Organization and Cell Motiliy by Phosphorylating the Actin Cross-Linking Protein EPLIN.. Mol Cell Biol.

[29] Clarke DM, Brown MC, LaLonde DP, Turner CE (2004). Phosphorylation of actopaxin regulates cell spreading and migration.. J Cell Biol.

[30] Ito S, Takahara Y, Hyodo T, Hasegawa H, Asano E (2010). The roles of two distinct regions of PINCH-1 in the regulation of cell attachment and spreading.. Mol Biol Cell.

[31] Senga T, Miyazaki K, Machida K, Iwata H, Matsuda S (2000). Clustered cysteine residues in the kinase domain of v-Src: critical role for protein stability, cell transformation and sensitivity to herbimycinA.. Oncogene.

[32] Yamasaki Y, Ito S, Tsunoda N, Kokuryo T, Hara K (2007). SIRPalpha1 and SIRPalpha2: their role as tumor suppressors in breast carcinoma cells.. Biochem Biophys Res Commun.

[33] Kurata H, Thant AA, Matsuo S, Senga T, Okazak K (2000). Constitutive activation of MAP kinase kinase (MEK1) is critical and sufficient for the activation of MMP-2. Exp.. Cell Res.

[34] Obenauer JC, Cantley LC, Yaffe MB (2003). Scansite 2.0: Proteome-wide prediction of cell signaling interaction using short sequence motifs.. Nuc Acids Res.

[35] Sossey-Alaoui K, Li X, Cowell JK (2007). c-Abl-mediated phosphorylation of WAVE3 is required for lamellipodia formation and cell migration.. J Biol Chem.

[36] Taieb D, Roignot J, Andre F, Garcia S, Masson B (2008). ArgBP2-dependent signaling regulates pancreatic cell migration, adhesion, and tumorigenicity.. Cancer Res.

[37] Zhang X, Li C, Gao H, Nabeka H, Shimokawa T Rho kinase inhibitors stimulate the migration of human cultured osteoblastic cells by regulating actomyosin activity.. Cell Mol Biol Lett.

[38] Magdalena J, Millard TH, Machesky LM (2003). Microtubule involvement in NIH3T3 Golgi and MTOC polarity establishment.. J Cell Sci.

[39] Pawson T (1994). SH2 and SH3 domains in signal transduction.. Adv Cancer Res.

