# Supplementary figures and images for: Role of Palladin Phosphorylation by Extracellular Signal-Regulated Kinase in Cell Migration
Source: PLoS One. 2011 Dec 28;6(12):e29338. doi: 10.1371/journal.pone.0029338 (PMC3247243; doi:10.1371/journal.pone.0029338)

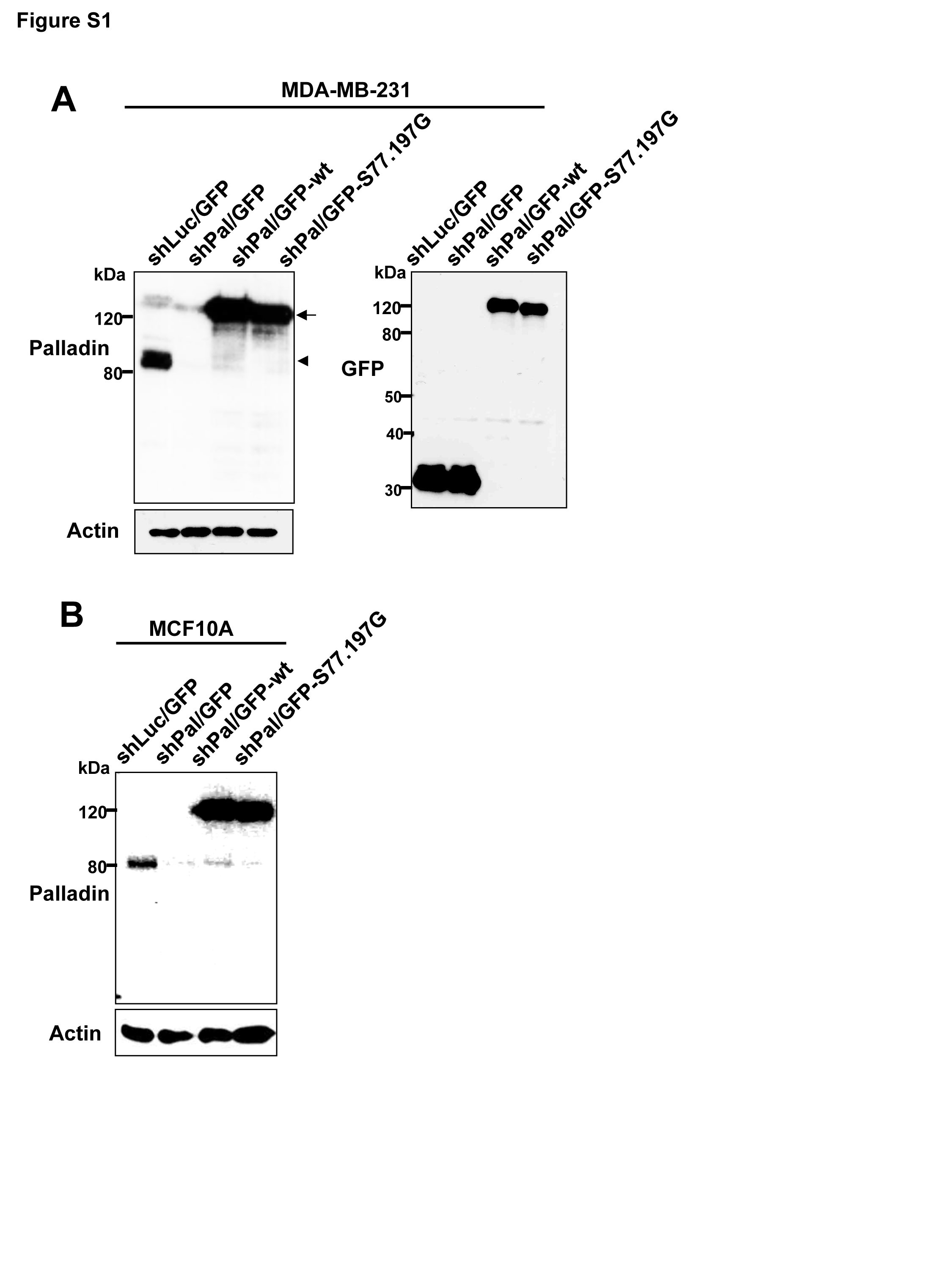

Supplement: Figure S1 — Expression of endogenous and exogenous palladin in each cell line. (A) Expression of endogenous palladin and exogenously expressed proteins in the indicated cell lines were examined by western blotting. An arrow indicates exogenously expressed palladin, and an arrowhead indicates endogenous palladin. (B) Endogenous palladin expression and exogenously expressed proteins in the indicated cell lines were examined by western blotting. (TIF) [file pone.0029338.s001.tif]

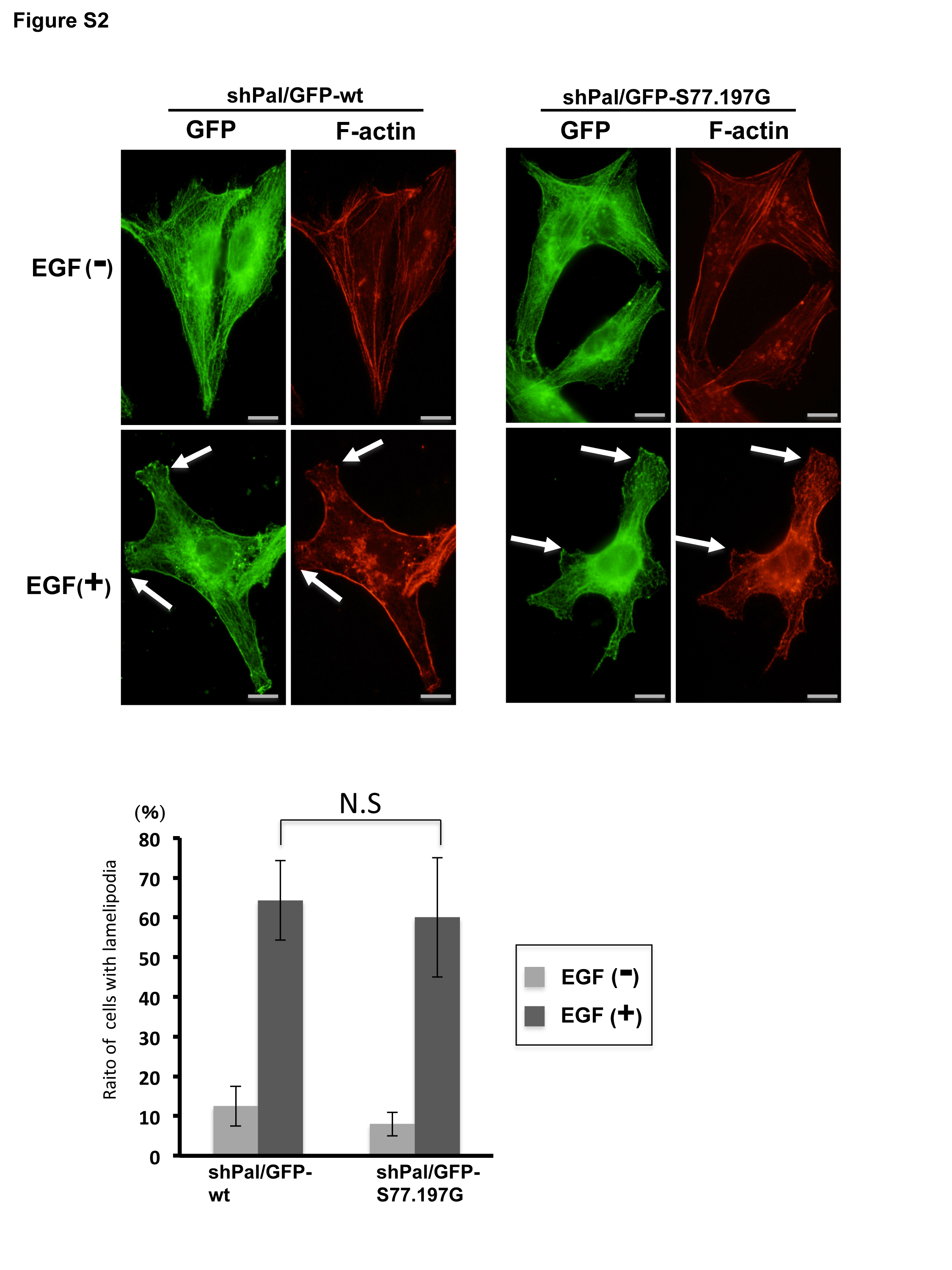

Supplement: Figure S2 — shPal/GFP-wt and shPal/GFP-S77.197G cells were serum-starved and stimulated with EGF for 5 min. Cells were fixed and immunostained with rhodamin-conjugated phalloidin. (Scale bar = 10 µm) The arrows indicate lamellipodia formation. The graph shows the ratio of cells with lamellipodia formation. Thirty cells were evaluated for lamellipodia formation and three independent experiments were performed. The data are shown as mean ±SD. (N.S; P>0.05). (TIF) [file pone.0029338.s002.tif]

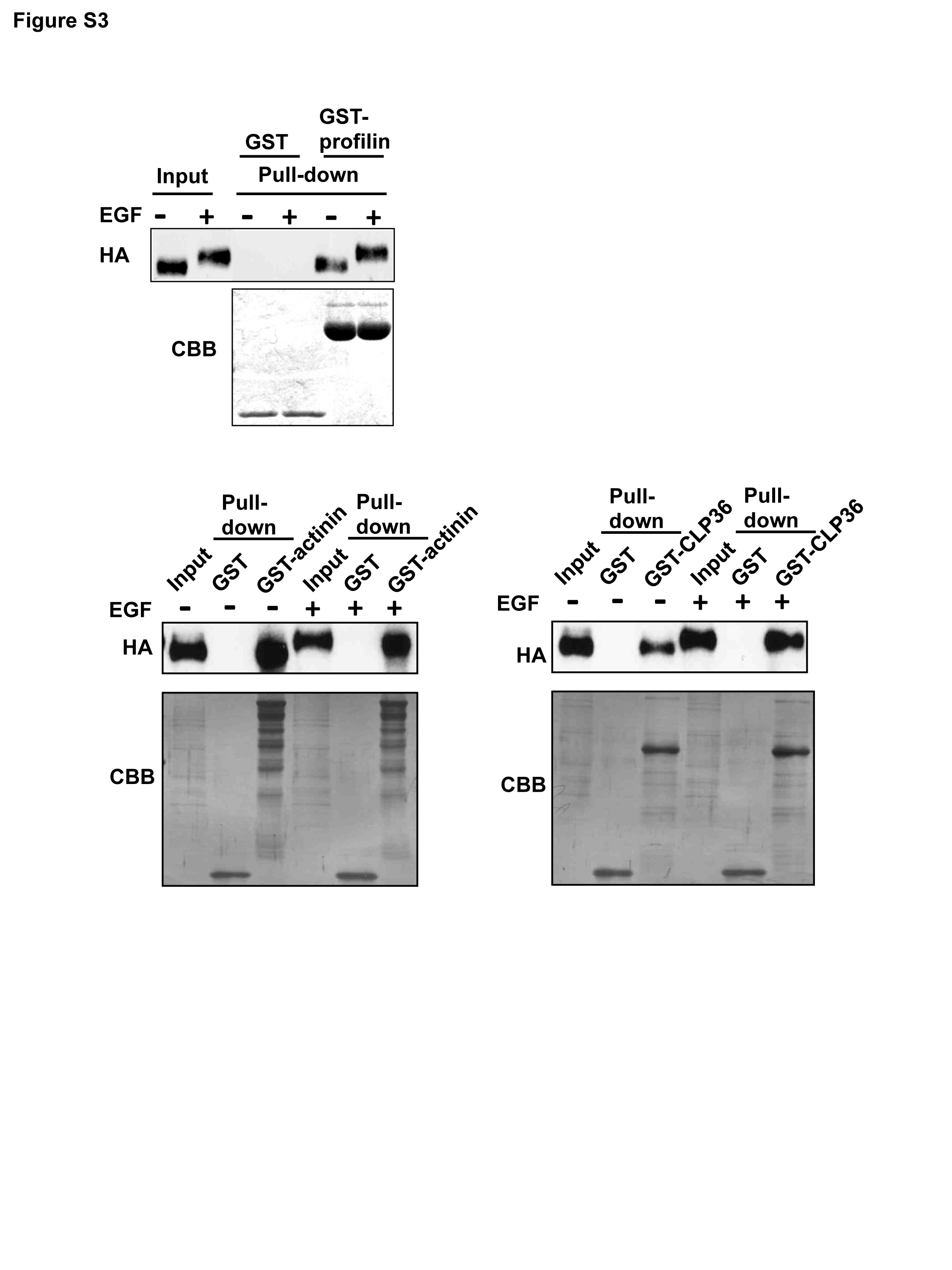

Supplement: Figure S3 — The phosphorylation of palladin does not affect the interaction with profilin, alpha-actinin, and CLP36. 293T/HA-wt cells were either stimulated or non-stimulated with EGF for 5 min, and cell lysates were affinity precipitated with the indicated recombinant proteins. The precipitate were immnoblotted with anti-HA antibody. Lower panels show the Coomassie blue staining of recombinant proteins. (TIF) [file pone.0029338.s003.tif]
